# Supplementary material for: Identification of Late Ripening Citrus Mutant, Ara-unshiu (Citrus unshiu), and Its Selectable Marker
Source: Plants (Basel). 2023 Sep 22;12(19):3355. doi: 10.3390/plants12193355 (PMC10574479; doi:10.3390/plants12193355)
Supplement: Supplementary file 1 [file plants-12-03355-s001.zip › plants-2597266-supplementary.pdf]

Table S1. Statistics of SNP detection in samples

Sample	No. of Total SNP	No. of Homozygous[1]	No. of Heterozygous[2]	No. of Etc.[3]
WT	1,198,650	8,208	572,811	617,631
Ara-unshiu	1,193,106	9,457	576,678	606,971

[1] The same SNP type showed with 90% of sample reads mapped to the reference genome

[2] The same SNP type showed with 40%~60% of sample reads mapped to the reference genome

[3] Not applicable as the homozygous / heterozygous

Table S2. Statistics of SNP classification by genome annotation

Sample	Total SNP	Classificated SNP[1]	IGR[2] & gene structur	Total	Homozygous	Heterozygous	Etc[3]
WT	1,198,650	1,083,127	intergenic	715,536	5,577	318,289	391,670
			genic region	367,591	1,338	209,859	156,394
			exon	164,743	605	94,330	69,808
			intron	212,665	776	120,993	90,896
Ara-unshiu	1,193,106	1,078,912	intergenic	712,344	6,265	320,049	386,030
			genic region	366,568	1,801	212,460	152,307
			exon	164,072	795	95,728	67,549
			intron	212,363	1,055	122,218	89,090

[1] Loci where genes are organized in reference gene annotation (gff) file and can be classified as genic/intergenic

[2] Intergenic region

[3] Not applicable as the homozygous / heterozygous

Table S3. Statistics of In/Del detection in samples

Sample	No. of Total In/Del		No. of Homozygous[1]		No. of Heterozygous[2]		No. of Etc.[3]	
	Total (Ins/Del)	Ins/Del	Total (Ins/Del)	Ins/Del	Total (Ins/Del)	Ins/Del	Total (Ins/Del)	Ins/Del
WT	172,259	88,264 / 83,995	3,751	1,600 / 2,151	57,930	29,136 / 28,794	110,578	57,528 / 53,050
Ara-unshiu	172,154	88,320 / 83,834	3,877	1,648 / 2,229	57,351	28,708 / 28,643	110,926	57,964 / 52,962

[1] The same SNP type showed with 90% of sample reads mapped to the reference genome

[2] The same SNP type showed with 40%~60% of sample reads mapped to the reference genome

[3] No applicapble as the homozygous / heterozygous

Table S4. Statistics of In/Del classification by genome annotation

Sample	Total In/Del	Classificated In/Del[1]	IGR[2] & gene structure	Total	Homozygous	Heterzygous	Etc[3]
WT	172,259	155,928	intergenic	110,566	2,429	35,781	72,187
			genic region	45,362	632	17,495	27,145
			CDS	3,799	201	1,339	2,259
			exon	11,128	295	4,149	6,684
			intron	35,441	359	13,863	21,219
Ara-unshiu	172,154	155,751	intergenic	110,398	2,494	35,410	72,494
			genic region	45,353	610	17,317	27,426
			CDS	3,757	177	1,250	2,330
			exon	11,105	278	4,048	6,779
			intron	35,448	354	13,762	21,332

[1] Loci where genes are organized in reference gene annotation (gff) file and can be classified as genic/intergenic

[2] Intergenic region

[3] Not applicable as the homozygous / heterozygous

If the reference gene position overlaps or due to the anti-sense form of the gene, the number of exons and introns may be counted as duplicates.

Table S5. Primer list used in AS-PCR

Forward Primers		Reverse Primrs	
Ara-SNP1 F	5- AATACGAGATGTTTTCATGCGAT-3	Ara-SNP1 R	5- AATACGAGATGTTTTCATGCGAG -3
Ara-SNP2 F	5- AACACTGAGCAAGTTATACTACCAT-3	Ara-SNP2 R	5- AACACTGAGCAAGTTATACTACCAG-3
Ara-SNP3 F	5- ACAGCCCTTCCCAACCAT-3	Ara-SNP3 R	5-CGCGTTTGGAGCTTTAAATTCC-3
Ara-SNP4 F	5-GAAACTGTTTACATGCCTGG-3	Ara-SNP4 R	5-CAGTTCCCTTGCAGTCTCTCA-3
Ara-SNP5 F	5- CTTGGACAGGATTGGAATGA-3	Ara-SNP5 R	5-CTGAAGGTTGTGTAGCAACT-3
Con-SNP4 F	5- CAGTCCAGTGGTTAAAGCAG-3	Con-SNP4 R	5-CAGTTCCCTTGCAGTCTCTCA-3
